# Specific Tax on Physicians

**Published:** 1877-07-20

**Authors:** 


SPECIFIC TAX ON PHYSICIANS.—At the late meeting of the Georgia Medical Association a committee was appointed to memorialize the Legislature to remove the specific tax on physicians. At a former meeting of the society, previous to the war, one of the editors of this journal—Dr. Word—with Dr. J. G. Westmoreland and Dr. Southgate, were appointed a committee to do the same thing. They accordingly drew up a paper, setting forth the reasons for relieving the physician from this burden, and made a strong appeal to the law-making power. The leading point urged was the fact, which they most conclusively proved, that medical men do more gratuitous work, and contribute more to the poor than all other classes combined. The amount of drugs given away to the indigent sick, which the State authorities or county fund ought to supply, not to mention the labor bestowed, was shown, by an estimate from an average county to be enormous, the single article of quinine amounting to over $50,000 per annum in the State. The memorial failed, as, in all probability, will be the case with this next effort. The memorial referred to was published in the Augusta *Medical Journal*, 1860.

